# Supplementary material for: Verapamil and its metabolite norverapamil inhibit the Mycobacterium tuberculosis MmpS5L5 efflux pump to increase bedaquiline activity
Source: Proc Natl Acad Sci U S A. 2025 Apr 17;122(16):e2426827122. doi: 10.1073/pnas.2426827122 (PMC12036985; doi:10.1073/pnas.2426827122)
Supplement: Supplementary file 1 — Appendix 01 (PDF) [file pnas.2426827122.sapp.pdf]

PNAS

Supporting Information for

Verapamil and its metabolite norverapamil inhibit the *Mycobacterium tuberculosis* MmpS5L5 efflux pump to increase bedaquiline activity

Adam J Fountain, Natalie JE Waller, Chen-Yi Cheung, William Jowsey, Michael T Chrisp, Mark Troll, Paul H Edelstein, Gregory M Cook*, Matthew B McNeil* and Lalita Ramakrishnan*

Gregory Cook
Email: g3.cook@qut.edu.au

Matthew McNeil
Email: matthew.mcneil@otago.ac.nz

Lalita Ramakrishnan
Email: lalitar@mrc-lmb.ac.uk

This PDF file includes:

Figures S1 to S3
Tables S1 to S2
SI References

Supporting Information

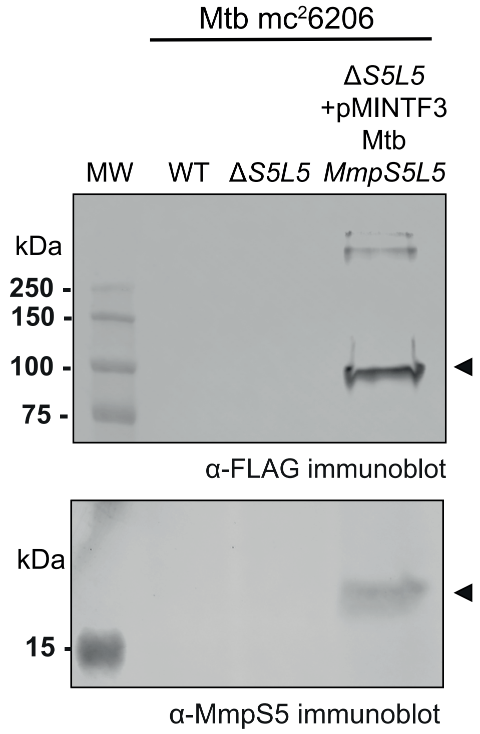

Fig S1. Immunoblot analysis of complemented strains:

20 µg whole cell lysate from Mtb mc^{2}6206, Δ*S5L5*::loxP and Δ*S5L5*::loxP attB$_{L5}$::pMINTF3 Mtb MmpS5L5 strains probed with mouse anti-FLAG or anti-Mtb MmpS5 (VFADDPEPFDPKVVC) antibody. Endogenous levels of MmpS5 in the wild-type strain are undetectable in whole-cell lysate.

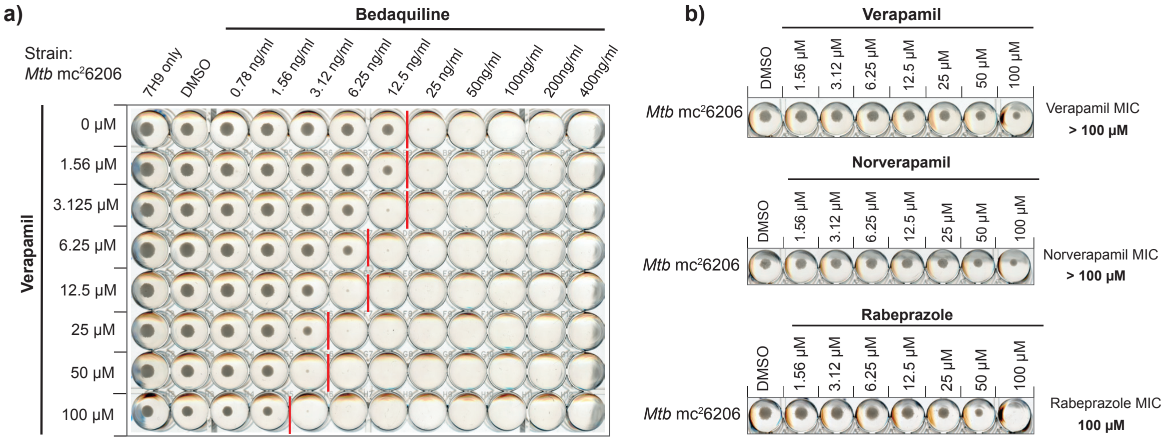

Fig S2. Representative checkerboard

(a) Representative checkerboard of BDQ–VER in Mtb mc^{2}6206. Red lines indicate drug MICs. (b) Verapamil, norverapamil and rabeprazole only columns, showing that verapamil/norverapamil MIC > 100 μM, whilst rabeprazole MIC = 100 μM.

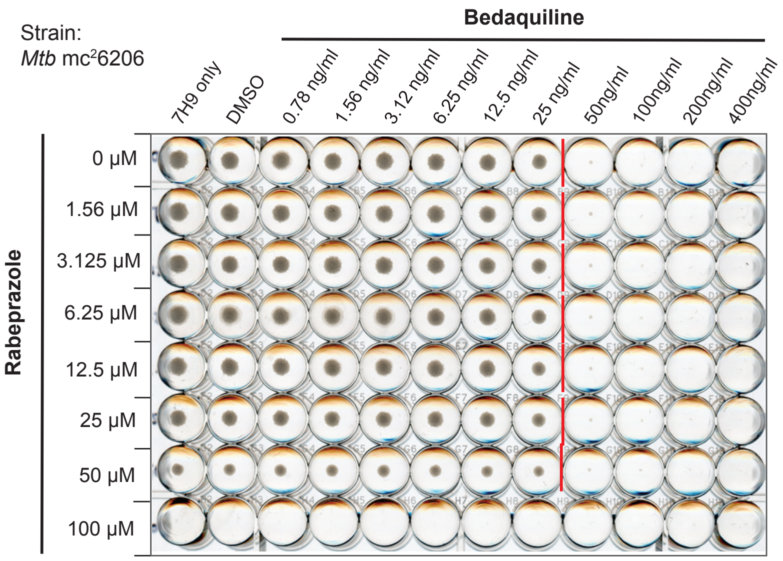

Fig S3. Rabeprazole does not potentiate bedaquiline MIC

(a) Representative checkerboard of BDQ–Rabeprazole in Mtb mc^{2}6206. Red lines indicate drug MIC. Rabeprazole has an MIC of 100 µM.

Table S1. Strains, plasmids and primers used in this study.

E.coli strains

Strain	Description	Source
MC1061	Cloning strain for CRISPRi plasmids	Lab strain
DH5-α	Cloning strain for knockout and complementation plasmids	Invitrogen

M. tuberculosis and *M. smegmatis* strains

Strain	Description	Source
M. tuberculosis $mc^2$6206	Drug-susceptible	(1)
M. tuberculosis $mc^2$6206 *Rv0678*^{G65GfsX10}	Isolated from bedaquiline containing media	(2)
M. tuberculosis $mc^2$6206 Δ*S5L5*::loxP	Unmarked MmpS5L5 KO strain	This study
M. tuberculosis $mc^2$6206 Δ*S5L5*::loxP, $attB_{L5}$::pMINTF3 Mtb MmpS5L5	Unmarked MmpS5L5 KO strain complemented with pMINTF3 Mtb MmpS5L5, Kan^R.	This study
M. smegmatis $mc^2$155	Transformable lab strain of *M.smegmatis*	(3)

Plasmids

Plasmid name	Description	Target sequence (Coding, 5'-3')	PAM (non coding 5'-3', NN…...)	Source
pJLR965	Cloning plasmid and negative control			Addgene #115163
pCi1214	CRISPRi knockdown of MmpL5	CTGAGCTTCACCCGACTGCC	ACAGAAC	This study
pCi1215	CRISPRi knockdown of MmpS5	GGTTCCGAAGGCATCTTGGT	AAAGAAA	This study
pNIT-ET-SacB-Kan	Episomal Isovaleronitrile-inducible expression of phage Che9c RecET enzymes for recombineering			Addgene #107692
pCre-SacB-Zeo	Episomal expression of Cre recombinase for excision of loxP-hyg-loxP casette			Addgene #107706
pKM342-S5L5KO	BsaI-domesticated pKM342 (Adapted from Addgene #71486) containing ~500bp sequences upstream and downstream of MmpS5L5 operon flanking a loxP-hyg-loxP cassette for recombineering.			This study
pMINTF3 Mtb MmpS5L5	L5 int, L5 attP integrating vector, constitutively expressing MmpS5L5-3xFLAG from pmyctetO promoter			This study

Oligonucleotides used in this study

Oligos for CRISPRi plasmid construction	
Oligo Name	Oligo Sequence
mmpL5_a_TB_Fcs	GGGAGGCAGTCGGGTGAAGCTCAG
mmpL5_a_TB_Rcs	AAACCTGAGCTTCACCCGACTGCC
mmpS5_a_TB_Fcs	GGGAACCAAGATGCCTTCGGAACC
mmpS5_a_TB_Rcs	AAACGGTTCCGAAGGCATCTTGGT

Table S2. Laboratory 1 and 2 MIC values

Laboratory 1 MIC values (Figures 1 and 3)

	Bedaquiline		Clofazimine		PBTZ-169		OPC-167832		TBAJ-876		TBAJ-587	
Strain	Geometric mean MIC (ng/ml)	Range (ng/ml)	Geometric mean MIC (ng/ml)	Range (ng/ml)	Geometric mean MIC (ng/ml)	Range (ng/ml)	Geometric mean MIC (ng/ml)	Range (ng/ml)	Geometric mean MIC (ng/ml)	Range (ng/ml)	Geometric mean MIC (ng/ml)	Range (ng/ml)
Mtb H37Rv mc^{2}6206	25	25	198	125–500	0.5	0.39–0.79	0.195	0.195–0.39	7.9	6.25–12.5	3.1	3.1
Mtb H37Rv mc^{2}6206 Rv0678(G65GfsX10)	141	100–200	500	500	3.12	3.12	0.491	0.39–0.78	44.5	25–50	12.5	12.5
Mtb H37Rv mc^{2}6206 Δ*S5L5*::loxP	6.25	6.25	31.2	31.2	0.39	0.39	0.195	0.195	3.12	3.12	1.56	1.56
Mtb H37Rv mc^{2}6206 Δ*S5L5*::loxP attB$_{L5}$::pMINTF3 *MmpS5L5*	84	50–100	250	250	1.56	1.56	0.491	0.39–0.78	14	12.5–25.0	6.25	6.25

Laboratory 2 MIC values (Figure 2)

	Bedaquiline		Clofazimine		PBTZ-169		TBAJ-876	
Strain	Geometric mean MIC (ng/ml)	Range (ng/ml)	Geometric mean MIC (ng/ml)	Range (ng/ml)	Geometric mean MIC (ng/ml)	Range (ng/ml)	Geometric mean MIC (ng/ml)	Range (ng/ml)
Mtb H37Rv mc^{2}6206	148	88-216	135.9	11.5–370	0.25	0.18–0.71	36.3	25.6–51.3
Mtb H37Rv mc^{2}6206 + MmpS5 gRNA	27	27	23.1	23.1	1.3*	0.9–1.78	6.4	6.4
Mtb H37Rv mc^{2}6206 + MmpL5 gRNA	19	13.5–27	16.4	11.5–23.1	0.9*	0.9	6.4	6.4
Mtb H37Rv mc^{2}6206 Rv0678(G65GfsX10)	796	436–1736	737.0	182–1439	1.6	0.7–2.9	290	102–411
Mtb H37Rv mc^{2}6206 Rv0678(G65GfsX10) + MmpS5 gRNA	75	54–108	5.8	5.8	0.18	0.18	9.1	6.4–12.8
Mtb H37Rv mc^{2}6206 Rv0678(G65GfsX10) + MmpL5 gRNA	38	27–54	2.9	1.4–5.8	0.18	0.18	9.1	6.4–12.8

Note: Differences in MICs between laboratories 1 and 2 are likely a result of differing methods of MIC determination. For clarity, MICs values are expressed in ng/ml rather than the standard unit of μg/ml. * - This experiment had higher MICs across all strains

SI References

1. Jain P, Hsu T, Arai M, Biermann K, Thaler DS, Nguyen A, et al. Specialized Transduction Designed for Precise High-Throughput Unmarked Deletions in Mycobacterium tuberculosis. mBio. 2014 Jun 3;5(3):e01245-14.

2. Waller NJE, Cheung CY, Cook GM, McNeil MB. The evolution of antibiotic resistance is associated with collateral drug phenotypes in *Mycobacterium tuberculosis*. Nat Commun. 2023 Mar 18;14(1):1517.

3. Snapper SB, Melton RE, Mustafa S, Kieser T, Jr WRJ. Isolation and characterization of efficient plasmid transformation mutants of *Mycobacterium smegmatis*. Molecular Microbiology. 1990;4(11):1911–9.
